# Fostering collective leadership to improve integrated primary care: lessons learned from the PriCARE program

**DOI:** 10.1186/s13690-024-01258-9

**Published:** 2024-02-22

**Authors:** Catherine Hudon, Mireille Lambert, Kris Aubrey-Bassler, Maud-Christine Chouinard, Shelley Doucet, Vivian R. Ramsden, Joanna Zed, Alison Luke, Mathieu Bisson, Dana Howse, Charlotte Schwarz, Donna Rubenstein, Jennifer Taylor

**Affiliations:** 1 Département de Médecine de Famille et de Médecine D’urgence, Université de Sherbrooke, 12e Avenue Nord, Sherbrooke, QC 3001 Canada; 2 Primary Healthcare Research Unit, Memorial University, Room 426, 4th Floor Janeway Hostel, Health Sciences Centre, 300 Prince Philip Dr, Saint John, NL Canada; 3 Faculté des Sciences Infirmières, Université de Montréal, 2375, Chemin de la Côte-Ste-Catherine, Montréal, QC Canada; 4 Department of Nursing and Health Sciences, University of New Brunswick, 100 Tucker Park Road, Saint John, NB Canada; 5 Department of Academic Family Medicine, University of Saskatchewan, 3311 Fairlight Drive, Saskatoon, SK Canada; 6 Department of Family Medicine, Dalhousie University, 1465 Brenton Street, Halifax, NS Canada

**Keywords:** Collective leadership, Case management, Complex healthcare intervention, Implementation

## Abstract

Case management (CM) is an intervention for improving integrated care for patients with complex care needs. The implementation of this complex intervention often raises opportunities for change and collective leadership has the potential to optimize the implementation. However, the application of collective leadership in real-world is not often described in the literature. This commentary highlights challenges faced during the implantation of a CM intervention in primary care for people with complex care needs, including stakeholders’ buy-in and providers’ willingness to change their practice, selection of the best person for the case manager position and staff turnover. Based on lessons learned from PriCARE research program, this paper encourages researchers to adopt collective leadership strategies for the implementation of complex interventions, including promoting a collaborative approach, fostering stakeholders’ engagement in a trusting and fair environment, providing a high level of communication, and enhancing collective leadership attitudes and skills. The learnings from the PriCARE program may help guide researchers for implementing complex healthcare interventions.

## Introduction

Healthcare interventions are often complex because of their multiple interacting components and the necessity to adapt them to different contexts. In addition, these interventions are dependent on the behaviors of individuals delivering and receiving the intervention, and could generate numerous outcomes [1–3]. Case management (CM), one of the most studied models of integrated care [4, 5], is recognized as a complex intervention which aims to assess, plan, facilitate, and coordinate care to meet the health care needs of patients and their families [6]. CM is an effective approach to improving clinical, patient-reported, and health system outcomes [5, 7–12].

The effectiveness of complex healthcare interventions such as CM is intrinsically linked to successful implementation [13]. However, implementing complex interventions often raises challenges for researchers and stakeholders (i.e. policy makers, managers, healthcare providers and patients). These challenges may be related to individuals’ skills, readiness, commitment, and leadership; professional and cultural norms; capability and resources of organizations; as well as policies, priorities, and laws [14, 15]. Pragmatic solutions are needed to address these and to optimize the implementation of complex healthcare interventions.

Adequate governance and leadership are required to address healthcare changes essential to the implementation of integrated care interventions [16, 17]. Previous studies have highlighted the potential for collective leadership to help overcome implementation challenges [18–20]. Collective leadership is an organizational culture where every member is encouraged to participate in decision-making, based on their strengths, experience and expertise [21, 22]. This type of leadership, calling for the involvement of everyone to work together toward common goals, improves staff engagement, quality of care, teamwork, and patient satisfaction [21–23]. Despite the potential of this approach, its real-word application is not often described in the academic healthcare literature. This paper aims to present pragmatic strategies of collective leadership based on lessons learned from PriCARE, a CM research program in primary care for people with complex care needs.

## The PriCARE program

The PriCARE program, a case study with a participatory approach detailed elsewhere [24], was conducted to implement and evaluate a CM intervention for people with complex care needs in primary care clinics in five Canadian provinces: New Brunswick, Newfoundland and Labrador, Nova Scotia, Quebec, and Saskatchewan. PriCARE is a patient-oriented research program in which patient partners, as full team members, participate in decision-making and research activities [25]. Health managers and providers were also solicited to collaborate on implementation activities according to their interest, expertise, and availability. CM was delivered by case managers (nurses or social workers) to people with complex care needs, focusing on four components: (1) evaluation of patient needs and preferences; (2) individualized care planning; (3) care coordination and support; and (4) self‐management support. A central coordinating team and local teams in each province, including academic researchers, primary care providers, and patient partners, oversaw the implementation of CM following steps proposed by Damschroder and colleagues [26].

(1) Engaging: The research team identified and involved key stakeholders, such as decision-makers, health managers and healthcare providers (e.g., primary care providers, case managers as well as managers in the clinic and the hospital). (2) Planning: The implementation was planned based on their needs and perspectives. The implementation strategies were flexible and could be adapted according to local needs and resources. Regular team meetings were scheduled for program follow up. Local team leaders developed and maintained regular contact with stakeholders to deliver appropriate information and support throughout the course of the program as well as to provide tools to facilitate the implementation. (3) Executing: The CM intervention was implemented in primary care clinics in the Fall of 2019. However, it was interrupted in March 2020 due to Covid-19 and started again in the winter of 2020. (4) Reflecting and evaluating: this paper is part of this phase. It is the result of the team’s reflective activity on challenges and lessons learned from the PriCARE program.

## Lessons learned from the PriCARE program

### Collective leadership at the organizational level

One key challenge in the implementation of complex healthcare interventions remains stakeholders’ commitment to the program and willingness to change their practice. Encouraging discussion and communicating and sharing a clear vision of the intervention were helpful in ensuring that everyone can understand its added value [20, 21].

Adoption of the proposed changes may be promoted by developing shared objectives with stakeholders, providing regular feedback, listening to local needs, and adapting the intervention accordingly while promoting a culture of collaboration [21, 27]. Stakeholders need to be consulted on key decisions, and it is important to provide the right balance of promoting the intervention without forcing change. In that sense, regular discussions should be promoted to communicate important information, share objectives, receive feedback, and discuss local adaptation of the intervention [21]. Team commitment is also promoted by developing a positive and trusting environment, recognizing added value of team members, encouraging innovation, and ensuring transparency [21].

In the PriCARE program, some physicians and other providers were reluctant to change their practice despite efforts made to encourage buy-in. To move everyone’s efforts in one direction, local team leaders encouraged managers to clearly highlight how patient-oriented services and CM were a priority for their organization. Local team leaders also created opportunities (e.g., meetings, documents, pamphlets) to familiarize providers with the program and discuss its advantages and disadvantages, resources and time needed, and the roles, responsibilities, and concerns of each stakeholder.

Local team leaders also developed a personal connection with stakeholders. Before the Covid-19 pandemic, they planned to meet health care providers at their clinics to explain the program, answer questions, and receive feedback. When this was no longer possible, they scheduled a telephone or online meeting. The central team leaders created a newsletter to keep stakeholders informed on program activities. As much as possible, health care providers were remunerated for the time they committed to the program. The central team leaders also recommended that case managers and providers be in the same clinic to improve teamwork.

### Collective leadership at the case manager level

It has been demonstrated that having the right person in the CM role can have a significant impact on the success of the intervention for people with complex care needs [28]. However, it can be difficult to identify the correct individual for the role. Special attention should be placed on the background, experience and expertise of the case manager, as well as personal skills and attitudes [22]. Individual characteristics such as self-confidence and supportiveness are important for collective leadership as well as the ability to relate with patients, show compassion, and possess a desire to involve people and family members in their care [20, 29]. A motivated person with strong interpersonal skills and leadership capacity should be sought for this position [22].

In the PriCARE program, the case managers were selected by the health managers and physicians in charge of the clinic, under the guidance of the local team leaders which highlighted that the CM role requires engagement, leadership, and interpersonal skills. After receiving training from the central team on the CM program, the case managers stayed in close contact with local team leaders for support and direction. The latter provided key information about the program, timely feedback, and quick adaptation to the program when requested. A Community of Practice (CoP) was also created to engage the case managers in collective learning where they shared their clinical challenges, successes, pitfalls, and solutions. This trusting environment enabled partnership among case managers. Throughout the program, local team leaders struggled with staff turnover. To ensure service continuity, the team agreed that a replacement plan was needed to determine who would fill the role, and what the transition plan would be. Going forward, such a plan should be developed earlier in the implementation process and, with this contingency in mind, recruiting and training more than one case manager would help support collective leadership (Table 1).

**Table 1:** Collective leadership principles associated with lessons learned in PriCARE to help overcome implementation challenges

Collective leadership principles	Lessons learned in PriCARE
Communicate a vision oriented on the provision of high-quality care at every level and agree on clear objectives	Disseminate largely that patient-centered care and CM is a priority for the organization Provide tools for facilitating communication and information about the program
Encourage dialogue, debate, and discussion among stakeholders	Organize regular discussions to communicate important information, share objectives, receive feedback, and discuss local adaptation of the intervention
Involve stakeholders in decision-making, listen, support, and empower them to lead the implementation	Consult stakeholders on key decisions, promote the intervention without forcing change, and adapt the intervention to local needs Train and support case managers Create a CoP for case managers
Foster stakeholders’ engagement by encouraging respect, recognizing everyone’s contribution, giving timely feedback, promoting equity and trust	Create a trusting environment, recognize the added value of stakeholders, encourage innovation, and ensure transparency
Promote collective leadership qualities and behaviors: compassion, support, commitment, desire to involve patients and their family in care;	Select case managers based on their engagement, leadership, and interpersonal skills

## Conclusions

Developing strategies of collective leadership early on can help overcome challenges arising from the implementation of complex healthcare interventions. Promoting a collaborative approach, fostering stakeholders’ engagement in a trusting and fair environment, providing a high level of communication, and enhancing collective leadership attitudes and skills should be considered when choosing strategies to support the implementation. The learnings from the PriCARE program could be used as a guide for other teams implementing complex healthcare interventions. Future studies could evaluate the effectiveness of strategies of collective leadership to the successful implementation of CM.

## Data Availability

All data generated or analysed during this study are included in this published article.

## Abbreviations

CM: case management
CoP: Community of Practice
